# Investigating the efficacy of purified tannin extracts from underutilized temperate forages in reducing enteric methane emissions in vitro

**DOI:** 10.1038/s41598-024-63434-9

**Published:** 2024-05-31

**Authors:** S. Verma, T. T. Akpensuen, S. Wolffram, J.-P. Salminen, F. Taube, R. Blank, C. Kluß, C. S. Malisch

**Affiliations:** 1 Grass and Forage Science / Organic Agriculture, Christian-Albrechts-University of Kiel, E24118 Kiel, Germany; 2 Department of Agroecology, Aarhus University, 8830 Tjele, Denmark; 3 Net Zero and Resilient Farming, Rothamsted Research, Okehampton, EX20 2SD UK; 4 Faculty of Agriculture, University of Jos, P.M.B 2084, Jos, Nigeria; 5 Animal Nutrition and Physiology, Christian-Albrechts-University of Kiel, E24118 Kiel, Germany; 6 Natural Chemistry Research Group, University of Turku, 20500 Turku, Finland

**Keywords:** Chemical biology, Plant sciences, Environmental sciences

## Abstract

The study investigated how the concentration and composition of purified tannin extracts, at various inclusion rates, affect the ruminal in vitro fermentation parameters. Tannin extracts were isolated from four different forage species: birdsfoot trefoil (*Lotus corniculatus)*, sulla *(Hedysarum coronarium),* big trefoil *(Lotus pedunculatus),* and salad burnet (*Sanguisorba minor*). Plants extracts were purified by Sephadex LH-20 gel chromatography and analyzed by UPLC–ESI–MS/MS. The results showed a large variation among the extracts from different species in terms of tannin composition and structural features. The extracts from salad burnet were dominated by hydrolysable tannins, comprising mainly ellagitannins. The extracts derived from sulla and big trefoil contained predominantly proanthocyanidins (PA), primarily composed of prodelphinidins with high mean degree of polymerisation (mDP). Birdsfoot trefoil extracts comprised procyanidin-rich PAs with low mDP. To determine whether the combined presence of tannins and flavonoid together lead to synergistic or antagonistic effects, the tannin extracts were incubated both with or without rutin at concentrations of 10, 20, and 30 g/kg DM, using a base substrate of perennial ryegrass (*Lolium perenne*, control). In general, all the tannin extracts decreased methane (CH_4_) production compared to the control, while no significant effect of rutin was observed on both gas (GP) and CH_4_ production, neither pure, nor in the simultaneous presence of tannins. The highest CH_4_ reduction (15%, at 30 g/kg DM) was observed from sulla and big trefoil extracts compared to control, but this was also supplemented with a concomitant reduction in GP (11%) indicating a reduction in feed digestibility. The extracts from birdsfoot trefoil and salad burnet reduced CH_4_ by up to 12% without significantly reducing GP, indicating the importance of tannin composition on ruminal fermentation.

## Introduction

One of the main challenges of the twenty-first century is to limit climate change and simultaneously attain food security for the rapidly growing population^1^. During the last 40 years the global per capita consumption of livestock products has doubled, and currently the livestock sector alone contributes to approximately 14–15% of the total anthropogenic greenhouse gas emissions^1,2^. If the current trends continue, agricultural methane (CH_4_) emissions have been projected to increase approximately by 30% in 2050 compared to the levels of 2010^3^. In 2019, ruminant enteric CH_4_ emissions were the largest contributor to agricultural emissions, amounting up to 2.2 billion tonnes of carbon dioxide equivalent (Gt CO_2_eq)^4^. Several reviews have emphasized that dietary manipulation is one of the most effective and pragmatic mitigation strategies that can concomitantly improve animal productivity as well as reduce CH_4_ emissions as they are directly linked to rumen fermentation patterns and resulting end-products^5–7^. Inclusion of bioactive forages, such as tannin containing forages, are deemed to be a particularly promising CH_4_ mitigation strategy due to their concomitant reduction of food-feed competition, and their beneficial effects on animal health and productivity^8–10^. For example, the inclusion of tannin-containing forages in grassland-based livestock production systems has the potential to reduce bloating incidences and parasitic burdens in animals^11,12^.

The benefits of tannins are derived from their molecular structure, which enables them to form stable complexes with feed components (such as protein or fiber) which can reduce the production of ammonia during ruminal fermentation and increase the fraction of rumen escape protein^13,14^. With the shift of the protein-tannin complexes to the abomasum, the complexes are prone to dissociate under acidic conditions, which makes them available for digestion in the small intestine and potentially improving N utilisation efficiency in ruminants^13,15^. In addition to reducing protein degradation in rumen, tannin-rich diets can reduce CH_4_ formation by reducing the extent of carbohydrate fermentation. This results in reduction in volatile fatty acids (VFA) production and/or shift to an increased molar proportion of propionate in the animal, which acts as an alternative sink for H_2_ produced during microbial metabolism, consequently leading to decreased enteric CH_4_ production^16,17^.

Despite the beneficial effects of tannins been widely recognised, several studies have shown contrasting results on their effect on N utilisation and CH_4_ production in animal^11,18,19^. The conflicting results on the effect of tannins on the animal productivity could arise from the limited consideration given to the chemical composition as well as structural attributes of tannins in many animal nutrition studies^20–22^. Simultaneously, with structural differences in tannins affecting the interactions with both gastrointestinal microbes and feed components, their large variability even within single species has been a major obstacle to predicting their bioactivity^9,23^.

Tannins are a heterogenous group of compounds with molecular weight ranging from 500 to 28,000 Daltons present in different plant species^20^. They can generally be grouped into condensed tannins, also known as proanthocyanidins (PAs), and hydrolysable tannins (HTs). Proanthocyanidins are one of the most abundant plant polyphenols and are formed by the condensation of flavan-3-ol monomers linked commonly by C4–C8 and C4–C6 interflavan bonds. They can be subgrouped as procyanidins (PCs) or prodelphinidins (PDs)^24–26^. The HTs, as the second class of terrestrial tannins, are further subdivided into gallotannins, ellagitannins (ETs) and simple gallic acid derivatives. Simple gallic derivatives are formed when the central polyol core, most often glucose, is esterified with gallic acid to form monogalloyl groups, while in gallotannins at least one of these groups needs to contain two or more galloyls linked to each other via an ester bond. Ellagitannins, on the other hand, are formed from pentagalloylglucose when two of the adjacent galloyl groups are linked to each other via a C–C bond to form the hexahydroxydiphenoyl (HHDP) group^26,27^. These HHDP groups can be further oxidized to form more complex monomeric structures that can be linked to each other to form oligomeric and even polymeric ellagitannins. Simultaneously, tens or even hundreds of different types of tannins generally co-exist in a plant cell with other specialized metabolites which can potentially exert synergistic or antagonistic effects, thereby exacerbating the high variability in their observed effects on ruminants^13^.

Flavonoids are a group of plant phenolics that have been shown to reduce CH_4_ production by modifying rumen fermentation, and methanogen and protozoal population in rumen^28,29^. Thus, the aim of this study was to assess the effect of purified tannin extracts with diverse structural characteristics both with or without rutin (flavanoid), to identify the impact of structural diversity and matrix effects on CH_4_ production in vitro*.* Specifically, the study aimed to attain the following objectives, (a) examine how purified tannin extracts from different species and concentrations impact in vitro CH_4_ and gas production (GP), (b) evaluate the effect of flavonoid addition on the measured in vitro fermentation parameters, and (c) analyse the shifts in CH_4_ production when both tannins and flavonoid are supplemented together.

## Results

### Chemical composition of the tannin extracts

Perennial ryegrass that was used as a base substrate for different treatments, had the following chemical composition, crude protein (CP): 139 g/kg DM; neutral detergent fiber (NDF): 231 g/kg DM; acid detergent fiber (ADForg): 237 g/kg DM; fat: 36.1 g/kg DM, ash: 80 g/kg DM, metabolisable energy (ME): 11.6, and net energy of lactation (NEL): 7.1. Tannin extracts from the four different species varied widely in terms of their chemical composition and structural attributes (Table 1). Total polyphenol and tannin concentration (PAs and HTs) in the extracts ranged from 117.8 to 370 mg/g DM and 100.6 to 367.4 mg/g DM respectively. Sulla exhibited highest polyphenol and tannin concentration across all the fractions ranging from 340.2 to 370.8 mg/g DM and 335 to 341.4 mg/g DM respectively. Sulla and big trefoil contained almost exclusively PAs as tannins (> 99%, Table 1), while birdsfoot trefoil contained predominantly PAs (on average 83%). Salad burnet was the only HT-rich source (on average 93%) in the present study. The structural characteristics of PA-rich sources were measured in terms of mean degree of polymerization (mDP) and share of PDs in the PA (PD%). Sulla and big trefoil tannin extracts can be categorized as PD-rich sources with high mDP (12.9–21.5), and birdsfoot trefoil was characterized as a PC-rich source with low mDP (7–14.7). Additionally, tannin concentration and chemical composition in the extracts were found to be influenced by the purification process. Previous studies have shown that the size of polymers (i.e. mDP) increased with increasing acetone concentration in the Sephadex LH-20 eluent^30,31^. Thus, three different acetone concentrations of 30, 50 and 80% acetone were selected separately as the fractions F1 (30%), F2 (50%) and F3 (80%). This could partially be seen in our results for the PD-rich extracts from sulla and big trefoil, where the F3 extracts consisted of higher mDP and PD% compared to the F2 fraction. The amount of tannins extracted from the fraction F1 (eluted with 30% acetone, less than 0.15 g) from the different species was insufficient to perform in vitro analyses. Thus, further analyses were performed on the fractions extracted only with the F2 and F3 fractions.

**Table 1:** Chemical composition of tannin extracts in different fractions (details see below) after Sephadex LH-20 purification. F1: fraction eluted with 30% acetone; F2: fraction eluted with 50% acetone; F3: fraction eluted with 80% acetone; TP: Total polyphenols; TT: Total tannins; TF: Total Flavonoids, HTs: Hydrolysable tannins; PA: Proanthocyanidins; mDP: mean degree of polymerization; PD%: prodelphinidin percentage.

Species	Fraction	TP (mg/g)	TT (mg/g)	TF (mg/g)	HTs (mg/g)	PA (mg/g)	mDP	PD %
Birdsfoot trefoil	F1	117.8	100.6	17.2	12.1	88.6	7.8	48.3
Birdsfoot trefoil	F2	239.8	229.1	10.7	39.6	189.6	14.7	51.3
Birdsfoot trefoil	F3	220.0	208.5	11.5	46.3	162.3	13.9	49.4
Sulla	F1	340.2	335.0	5.2	1.1	333.9	12.9	91.1
Sulla	F2	370.8	367.4	3.4	0.8	366.6	17.7	92.3
Sulla	F3	341.6	341.4	0.2	0.6	340.9	24.7	92.6
Big trefoil	F1	264.2	258.1	6.1	0.5	257.5	15.3	86.5
Big trefoil	F2	339.4	331.6	7.8	0.5	331.1	14.7	83.2
Big trefoil	F3	293.3	292.2	1.1	0.9	291.3	21.5	88.0
Salad burnet	F1	172.7	165.5	7.2	151.6	13.9	3.9	41.1
Salad burnet	F2	183.6	182.1	1.5	166.9	15.2	6.7	59.1
Salad burnet	F3	179.1	178.4	0.7	171.1	7.3	4.3	36.9

### Effect of tannin source and concentration on total gas and methane production

The tested tannin extracts were found to reduce CH_4_ production compared to the tannin free control (Table 2). Methane production was found to be significantly affected by plant species, fraction and concentration in which extracts were added to the substrate (*p* < 0.001). Plant species (*p* < 0.05) and inclusion rate (*p* < 0.001) significantly influenced GP, whereas type of fraction did not significantly affect the GP. The maximum reduction in GP compared to control was observed at the concentration of 3% tannin extracts from sulla and big trefoil, which reduced GP by 10 and 12% respectively. Similar to GP which is used as a proxy for DM digestibility^32^, we found that the only significant reduction in OMD, ME and NEL was observed for sulla (30 g/kg DM) and big trefoil (20 and 30 g/kg DM). Simultaneously, both source (*p* < 0.05) and concentration (*p* < 0.001) were found to significantly impact these parameters.

**Table 2:** Mean values of in vitro fermentation parameters measured in extracts from various species and their fractions at different concentrations, in comparison to perennial ryegrass (control). Values within parentheses represent the standard error of the mean (SEM). Within a column, an asterisk (*) denotes significant differences (*P* < 0.05) compared to perennial ryegrass.

Species	Concentration (g/kg DM)	Fraction	Gas production (ml/200 mg DM)	Methane production (ml/200 mg DM)	Methane percentage in total gas (ml/200 mg DM)	In vitro organic matter digestibility (%)	Metabolisable energy (MJ/kg DM)	Net energy of lactation (MJ/kg DM)
Perennial ryegrass	0		65.3 (1.4)	13.5 (0.1) *	20.7 (0.5) *	79.7 (1.3)	10.9 (0.2)	6.8 (0.2)
Birdsfoot trefoil	10	F2	62.1 (1.4)	13.1 (0.2) *	21 (0.3)	76.9 (1.3)	10.5 (0.2)	6.5 (0.1)
Birdsfoot trefoil	20	F2	62 (1)	12.6 (0.1) *	20.3 (0.3) *	76.7 (0.9)	10.4 (0.2)	6.5 (0.1)
Birdsfoot trefoil	30	F2	61.9 (1.9)	12.8 (0.2) *	20.8 (0.4) *	76.7 (1.7)	10.4 (0.3)	6.5 (0.2)
Birdsfoot trefoil	10	F3	61.2 (2.1)	12.4 (0.2) *	20.2 (0.4) *	76.1 (1.9)	10.3 (0.3)	6.4 (0.2)
Birdsfoot trefoil	20	F3	60.5 (1.7)	12.2 (0.1) *	20.2 (0.4) *	75.5 (1.5)	10.2 (0.2)	6.3 (0.2)
Birdsfoot trefoil	30	F3	62.6 (0.8)	13.1 (0.4)	20.9 (0.6)	77.3 (0.8)	10.5 (0.1)	6.5 (0.1)
Sulla	10	F2	60.7 (1.5) *	12.4 (0.3) *	20.5 (0.2)	75.6 (1.4)	10.3 (0.2)	6.3 (0.2)
Sulla	20	F2	59.6 (0.7)	12 (0.3) *	20.2 (0.5)	74.6 (0.7)	10.1 (0.1)	6.2 (0.1)
Sulla	30	F2	64.8 (1.5)	12.8 (0.4)	19.7 (0.5)	79.2 (1.3)	10.9 (0.2)	6.8 (0.2)
Sulla	10	F3	58.4 (1.1)	12.7 (0.1)	21.8 (0.3)	73.6 (1)	9.9 (0.2)	6.1 (0.1)
Sulla	20	F3	56 (1.3) *	11.7 (0.5) *	21 (0.7)	71.4 (1.2) *	9.6 (0.2) *	5.8 (0.1) *
Sulla	30	F3	62.9 (1.2)	12.5 (0.1) *	19.9 (0.3)	77.6 (1.1)	10.6 (0.2)	6.6 (0.1)
Big trefoil	10	F2	59.8 (1.3)	12.4 (0.2) *	20.8 (0.4)	74.8 (1.1)	10.1 (0.2)	6.2 (0.1)
Big trefoil	20	F2	57.5 (2.1)*	11.7 (0.2) *	20.4 (0.6)	72.8 (1.9)	9.8 (0.3)	6 (0.2)
Big trefoil	30	F2	60.7 (1.3)	12.3 (0.2) *	20.3 (0.2)	75.6 (1.1)	10.3 (0.2)	6.3 (0.1)
Big trefoil	10	F3	55.1 (2.1) *	11.6 (0.2) *	21.1 (0.6)	70.7 (1.9) *	9.5 (0.3) *	5.8 (0.2) *
Big trefoil	20	F3	56.9 (1.4) *	11.6 (0.2) *	20.4 (0.4)	72.3 (1.2)	9.7 (0.2) *	5.9 (0.1) *
Big trefoil	30	F3	64.5 (2.2)	12.7 (0.2)	19.8 (0.6)	79 (1.9)	10.8 (0.3)	6.7 (0.2)
Salad burnet	10	F2	62.5 (2)	12.4 (0.3) *	19.8 (0.4) *	77.2 (1.8)	10.5 (0.3)	6.5 (0.2)
Salad burnet	20	F2	62.7 (2)	12 (0.5) *	19.2 (0.9) *	77.4 (1.8)	10.6 (0.3)	6.6 (0.2)
Salad burnet	30	F2	64.5 (1.3)	12.9 (0.3)	20 (0.4) *	79 (1.1)	10.8 (0.2)	6.7 (0.1)
Salad burnet	10	F3	61.4 (1.4)	12.1 (0.5) *	19.7 (0.5) *	76.2 (1.3)	10.4 (0.2)	6.4 (0.1)
Salad burnet	20	F3	60.5 (1.9)	11.9 (0.4) *	19.8 (0.8) *	75.5 (1.7)	10.2 (0.3)	6.3 (0.2)
Salad burnet	30	F3	60.5 (1.9)	11.9 (0.4) *	19.8 (0.8) *	79.9 (1.7)	11.8 (0.3)	7.4 (0.2)
	*P-*values							
	Source		< 0.05	< 0.001	< 0.001	< 0.05	< 0.05	< 0.05
	Concentration		< 0.001	< 0.001	0.22	< 0.001	< 0.001	< 0.001
	Fraction		< 0.01	< 0.001	0.69	< 0.01	< 0.01	< 0.001
	Source:Concentration		0.37	0.067	0.56	0.65	0.65	0.65
	Soure: Fraction		0.24	0.86	0.91	0.73	0.73	0.73
	Fraction:Concentration		0.4	0.42	0.54	0.64	0.64	0.64
	Source:Fraction:Concentration		0.79	0.46	0.33	0.47	0.47	0.47

For all the treatments, CH_4_ percentage in the gas produced (MP) was found to be lower compared to control (grass substrate, 21.6%). No significant effect of concentration and fraction of the extracts was observed on MP, and was found to be significantly affected only by the species of the tannin extracts. In contrast to the extracts from sulla and big trefoil, the tannin extracts isolated from birdsfoot trefoil and salad burnet consistently demonstrated significant reductions in MP (up to 11%) when compared to the control group, while exhibiting non significant reductions in GP.

**Figure 1:**
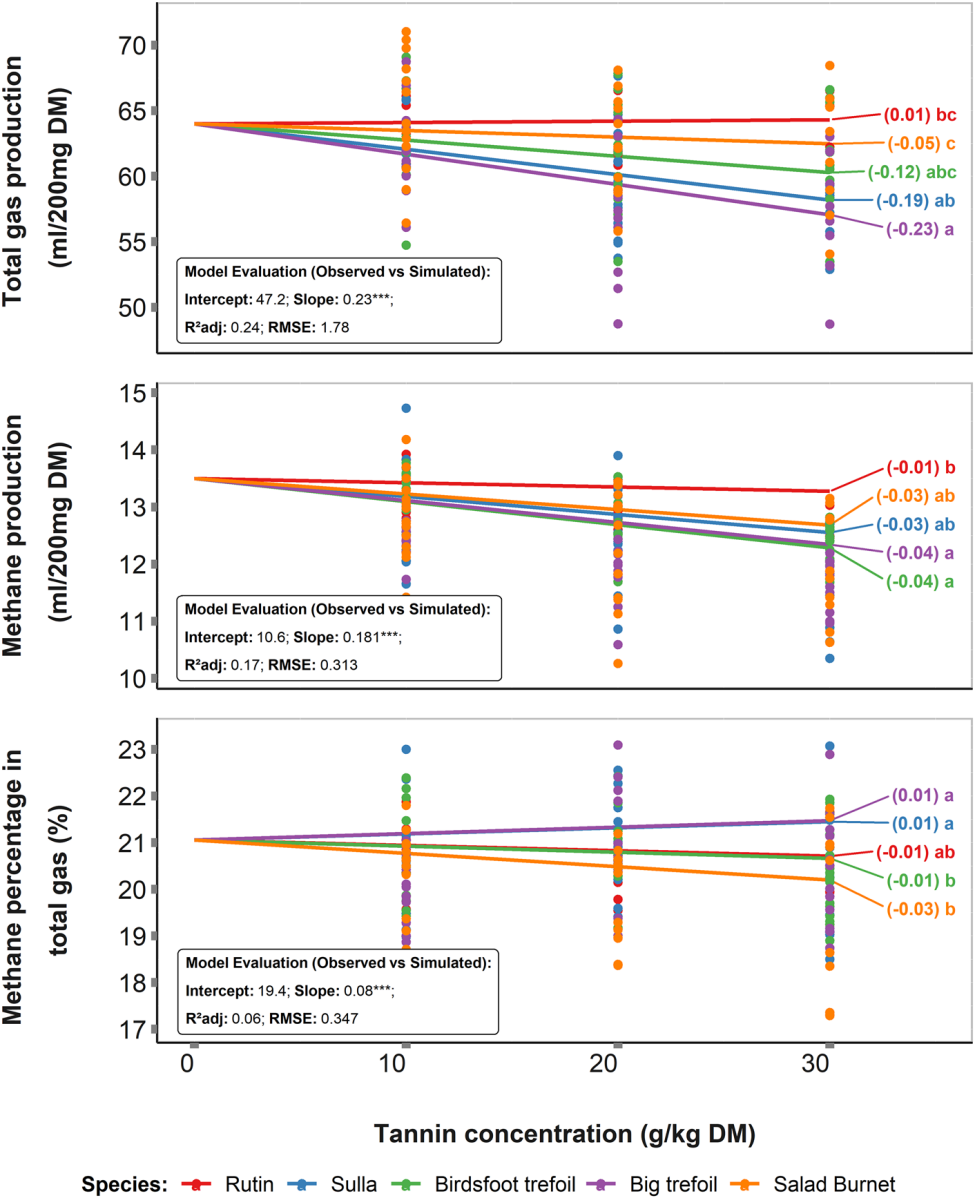
Measured fermentation parameters (Total gas production (mL/200 mg DM), Methane production (mL/200 mg DM) and Methane percentage in total gas (%)) after 24 h in vitro-rumen fermentation of the extracts from different tannin species and rutin mixed in increasing proportions with per forage.

Based on the measured values, we modelled the response of tannin inclusion rate from different plant species on GP, CH_4_ production, and MP (Fig. 1). We found that the model for GP was better suited to explain the variability (R^2^adj = 0.24) compared to the models for CH_4_ production (R^2^adj = 0.17), and MP (R^2^adj = 0.06). The highest GP reduction was predicted for big trefoil and sulla with the slopes of − 0.23 and − 0.19 respectively. However, the greatest CH_4_ reduction was predicted from birdsfoot trefoil and big trefoil with the slope of -0.04. Simultaneously, the model predicted an increment of MP for both big trefoil and sulla, whereas the highest reduction in MP was predicted for salad burnet (slope = 0.03).

### Effect of incorporating rutin in grass substrate on the in vitro fermentation profile

The study used rutin to assess potential synergistic or antagonistic matrix effects between flavonoids and tannins on in vitro CH_4_ and GP in this study. In contrast to the treatments with tannin extracts, rutin addition itself did not affect the fermentation parameters, and neither GP, nor CH_4_ production and MP differed significantly from the control (*P* < 0.05). The estimated parameters i.e. OMD, NEL and ME were also found to adhere to the GP pattern and were not significantly different than the control. However, a decrease in CH_4_ production compared to control was observed, which also resulted in lower MP from the samples incubated with rutin. These trends are further illustrated in Table 3. Additionally, we examined the impact of rutin addition with tannin extracts on the measured fermentation parameters. When rutin and tannin extracts were introduced together into the substrate, a general pattern of increased GP and CH_4_ production was emerged, in contrast to the treatment containing only tannin extracts at inclusion rate of 20 g/kg DM. However, this change was only significant for the big trefoil extracts (*P* < 0.05) (Table 3).

**Table 3:** Effect of rutin addition on different tannin extracts on methane production (ml/200 mg DM), gas production (ml/200 mg DM), and methane percentage in total gas (%). Values within parentheses represent the standard error of the mean (SEM).

Extract	Concentration (g/kg DM)	Gas production (ml/200 mg DM)	Methane production (ml/200 mg DM)	Methane percentage in total gas (%)	In vitro organic matter digestibility (%)	Metabolisable energy (MJ/kg DM)	Net energy of lactation (MJ/kg DM)
Rutin	10	63.9 (0.8) b	12.6 (0.1) b	20 (0.5) b	78 (1.4) b	10.7 (0.2) b	6.6 (0.2) b
Rutin	20	64.2 (1.2) b	12.8 (0.2) ab	20.8 (0.4) ab	76.7 (1.7) ab	10.4 (0.3) ab	6.5 (0.2) ab
Rutin	30	63.4 (1.6) b	13 (0.2) b	19.9 (0.4) b	79.7 (1.4) b	10.9 (0.2) b	6.8 (0.2) b
Birdsfoot Trefoil	10	61.9 (1.9) ab	12.4 (0.2) ab	20.2 (0.4) ab	76.1 (1.9) ab	10.3 (0.3) ab	6.4 (0.2) ab
Birdsfoot Trefoil + Rutin	10 + 10	65.3 (1.6) b	12.8 (0.4) b	19.7 (0.5) b	79.2 (1.3) b	10.9 (0.2) b	6.8 (0.2) b
Birdsfoot Trefoil	20	61.2 (2.1) ab	13.1 (0.2) b	20.8 (0.5) b	77.9 (1.3) b	10.6 (0.2) b	6.6 (0.2) b
Sulla	10	64.8 (1.5) b	12.6 (0.1) ab	21.2 (0.4) ab	74.3 (1) ab	10 (0.2) ab	6.2 (0.1) ab
Sulla + Rutin	10 + 10	63.3 (1.4) b	12.3 (0.2) ab	20.3 (0.2) ab	75.6 (1.1) ab	10.3 (0.2) ab	6.3 (0.1) ab
Sulla	20	59.2 (1.2) ab	12.2 (0.4) ab	20.9 (0.6) ab	73.6 (1.7) ab	9.9 (0.3) ab	6.1 (0.2) ab
Big trefoil	10	60.7 (1.3) ab	11.6 (0.2) a	21.1 (0.6) a	70.7 (1.9) a	9.5 (0.3) a	5.8 (0.2) a
Big trefoil + Rutin	10 + 10	58.5 (1.9) ab	12.9 (0.3) b	20 (0.4) b	79 (1.1) b	10.8 (0.2) b	6.7 (0.1) b
Big trefoil	20	55.1 (2.1) a	13.3 (0.1) b	20.8 (0.6) b	78.6 (1.8) b	10.7 (0.3) b	6.7 (0.2) b
Salad Burnet	10	64.5 (1.3) b	12.1 (0.5) ab	19.7 (0.5) ab	76.2 (1.3) ab	10.4 (0.2) ab	6.4 (0.1) ab
Salad Burnet + Rutin	10 + 10	64 (2.1) b	13.3 (0.1) b	20.8 (0.6) b	78 (1.8) b	12 (0.2) ab	7.1 (0.2) b
Salad Burnet	20	61.4 (1.4) ab	12.1 (0.5) ab	19.7 (0.5) ab	75.6 (1.3) ab	12.4 (0.3) b	6.8 (0.1) ab

Different lowercase letters represent the significant differences across extracts within a column.

## Discussion

The identification of key factors that determine the biological activity of tanniferous forages on CH_4_ production in ruminants has generally been made very difficult, due to the large number of confounding factors, such as the digestibility of the forage itself, the co-presence of flavonoids, or the large diversity in structural characteristics of the tannins. So far, reported results on the antimethanogenic activity of tannin containing forages have been quite variable and grouping them based only on HTs or PAs have led to contrasting results^9,23^. This suggests that structural properties of tannins contribute to their biological activity. However, limited studies are available which focus on elucidating the relationship between the structural attributes of tannins and their antimethanogenic activity. Consequently, in this study we explored these relationships by assessing the antimethanogenic activity of purified tannin fractions from four different species while using same substrate for all the samples.

### Structural characterisation of purified tannin extracts

Tannin extracts from sulla, salad burnet, big trefoil, and birdsfoot trefoil exhibited a diversity in tannin concentration and their structural characteristics. The tannin composition of the extracts in the present study aligns with previous findings^25,33^. Tannins extracted from plant material using aqueous acetone also contains various non-tannin constituents, such as sugars, monomeric flavonoids and their glycosides, phenolic acids, proteins, and lipids^26^. In order to remove these impurities, tannin extracts were purified using Sephadex LH-20 gel chromatography which has been widely used to purify initial tannin extracts and is known to have a high recovery rate^30^. In general, PAs comprising mainly PCs tend to have lower mDP values as seen for birdsfoot trefoil extracts in this study, and PD-rich PAs usually occur as mixtures of large polymers with high mDP values which was found to be true for sulla and big trefoil^10^. This could likely be the reason of higher PD% in the latter fractions of these extracts.

### Tannin extracts from birdsfoot trefoil and salad burnet had no negative effect on in vitro substrate digestibility

In general, tannin extracts reduced CH_4_ production compared to the tannin free control, however, the reduction was not statistically significant across all the tested extracts. At an inclusion rate of 10 g/kg DM, tannin extracts were found to decrease CH_4_ production without significantly reducing GP compared to control. With increase in tannin concentration to 30 g/kg DM, a reduction in GP with CH_4_ reduction was observed. This concomitant reduction of GP and CH_4_ production was more prominent for big trefoil and sulla extracts, a trend verified by their significantly steeper negative slopes for GP. However, this reduction from the purified extracts was lower compared to the reduction observed from the leaf samples of sulla and big trefoil (PA: 1.7–2.3% DM) compared to lucerne, as they reduced GP and CH_4_ production by up to 42 and 48% respectively^34^.

One possible reason for lower reduction of GP and CH_4_ production in the current study compared to the aforementioned study could be the choice of reference. In the current study, perennial ryegrass was utilised as a control which was found to have a high energy content (NEL: 7.1 MJ kg DM) and digestibility (78%). The GP from perennial ryegrass control (62.9 ± 0.8 mL/200 mg DM) was comparable to the average GP of the concentrate standard (65.1 mL/200 mg DM) used in our study after 24 h of incubation. The low antimethanogenic potential from the tannin extracts used in this study could be a result of the highly digestible substrate used in our mixtures, and larger antimethanogenic effects could potentially be achieved if the feed was of lower quality, thereby resulting in higher CH_4_ emissions to begin with^35,36^. Highly digestible forages have previously been shown to result in low CH_4_ emissions, due to their efficient breakdown in the rumen^2,7^. In a study by Battelli et al.^37^ observed that the CH_4_ reduction potential of quercetin differed widely depending on the basal feed (maize silage vs grass silage) which was used. These differences could be ascribed to the variation in the chemical composition of the basal feeds, maize and grass silage. The presence of starch in the maize silage could favor an increased propionate production which acts as an H_2_ sink and resulting in decreased enteric CH_4_ production^7,16^. Furthermore, inclusion of quercetin with maize silage may have imparted synergistic decrease in CH_4_ production compared to its addition with grass silage.

With regards to the tannin addition rate, a meta-analysis conducted by Berça et al.^38^ comprising wide range of tannin containing forages deduced that diets including PA concentrations less than 124 g/kg DM had no negative effect of dry matter intake by the animal, indicating that all concentrations utilized in this experiment would be far below the limit where palatability is reduced. The observed reduced OMD in the presence of sulla and big trefoil extracts (at 20 and 30 g/kg DM) can be ascribed to their high polymer size as well as dominance of PD-rich PAs, in contrast with birdsfoot trefoil. Several studies have shown that in addition to concentration, PAs with high PD% and polymer size (mDP value) can reduce protein degradation more effectively compared to the forages dominated with PC-rich PAs^10,39–41^.

Inclusion of these forages in pasture-based livestock production systems especially in temperate regions is quite promising as these forages are non-bloating and can be grown under wide range of climatic conditions^42^. The conventional legumes such as white clover and alfalfa, often exceed the required dietary CP concentrations (> 20% DM) for animals which can lead to inefficient N utilization in animals, and increased incidences of bloat thereby, negatively impacting both, animal health and productivity^8,43^. It is important to highlight that depending on the binding strength of PAs in PA-protein complexes, they have the potential for enhancing the flow of feed by-pass protein and there is a possibility of protein being available for digestion post-ruminally. These complexes are assumed to be dissociated under acidic conditions in the abomasum, making them available for digestion in the intestine^13,44^. This was further corroborated by an in vivo study by Lagrange et al.^44^ which demonstrated that beef heifers grazing solely on birdsfoot trefoil resulted in 40% higher average daily gain compared to animals grazing on lucerne while also reducing total urinary N compared to lucerne. A study by Orlandi et al.^45^, has also shown that tannin extracts from *Acacia mearnsii* increased the amino acid flux in to the duodenum in Holstein steers by 30% compared to the control and increasing the amino acid flow in the duodenum. The low correlation between the tannin chemical composition and MP further illustrates the functional diversity of tannins in the forages and a straightforward relationship between concentration and their bioactivity would not hold true for all the plant species.

### Salad burnet as HT rich species exhibits greater promise for reducing methane production in comparison to PA-rich species

Several studies have assessed the effect of HT-rich extracts on CH_4_ emissions. However, these studies have generally focused on a limited number of commercial tannin extracts from chestnut (*Castanea sativa*), sumach (*Rhus typhina*) and valonea (*Quercus aegilops*)^46,47^. Compared to aforementioned PA containing extracts, the reduction in GP was found to be much lower and non-significant (up to 3%) from HT-rich salad burnet extracts. However, these extracts showed a significant decrease in CH_4_ production (up to 12%). As a result, these extracts consistently demonstrated lowest MP among all the analysed tannin extracts, indicating the lowest CH_4_ emissions per unit of digestible dry matter. Similar results were observed in an in vivo study by Stewart et al.^18^, where salad burnet hay showed the lowest CH_4_ emitted per unit dry matter intake compared to birdsfoot trefoil and sainfoin (*Onobrychis viciifolia*) hays. Furthermore, studies by Jayanegara et al.^47^ and Hassanat and Benchaar^46^ also found lower reduction in GP when using HT-rich extracts compared to PA-rich extracts. This is likely due to the fact that PAs form stronger complexes with nutrients in comparison to HTs due to their higher degree of polymerization, making their degradation in ruminal environment much more difficult^47^. Additionally, higher ruminal fermentation under the supplementation of HT extracts compared to PAs could also be related to their higher susceptibility to degradation upon hydrolysis into monomers and other derivatives, such as gallic acid, ellagic acid, valoneic acid dilactone and pyrogallol, which could reduce their bioactivity. However, hydrolysis products obtained after HT degradation may lead to their enhanced antimethanogenic activity^48,49^.

Compared to PAs, the effect of HTs on CH_4_ reduction is more consistent although the precise mechanism of their action remains unclear. In a 60-day feeding trial by Liu et al.^50^, chestnut tannins reduced CH_4_ production by 25% in sheep without incurring any negative effects on their growth performance. This was attributed to a significant reduction in methanogen and protozoal population in the rumen with the addition of chestnut tannins in the study. On the contrary in the study by Salami et al.^48^, no effect of chestnut tannin supplementation on the community structure or abundances of methanogen and protozoa population was observed. This variation in the responses to HT tannin supplementation could arise from the variation in the individual composition of microbiota that colonizes the gastrointestinal tract of the different animals and the duration of tannin exposure^51^. Additionally, factors such as plant origin, tannin concentration and molecular structure, and dosage can result in their variable antimethanogenic effects^23,52^. This suggests that the effect of a single HT source could not be generalized to all HT-rich sources. This was also observed in the study by Hassanat and Benchaar^46^ where at the same concentration (50 g/kg) valonea extracts reduced ruminal CH_4_ production (10%) without any significant negative impact on total VFA concentration (i.e. feed fermentation, 3%) compared to control whereas inclusion of chestnut tannins at the same concentration results in concurrent reduction of both CH_4_ (13%) and total VFA concentration (8%). Additionally, most studies commonly analyze the bioactive effects of HTs on ruminal fermentation using either commercially available extracts with undefined chemical compositions or unpurified plant extracts. This renders it difficult to establish conclusive links between different structural characteristics of HTs and their bioactivity. Furthermore, it is possible that rumen microbial population could get adapted to HTs in the feed in the long-term. In a long-term (190 days) in vivo study by Wischer et al.^53^, even though in the beginning of the experiment the extracts reduced CH_4_ by upto 10% in the first week, no significant antimethanogenic effect of chestnut and valonea extracts (HT-rich) was observed after 2 weeks of supplementation. Such studies are very important for the nutritional assessment of additives as studies assessing the long-term effect of tannin supplementation on animal productivity as well as their CH_4_ reduction potential are sparse, especially in vivo. It is worth noting that when supplied in high concentration, HT-rich extracts could also be detrimental to animal health. Oak leaf extracts, which are rich in HTs could incur toxic effects on the cattle as they would readily degrade into absorbable low molecular weight compounds such as catechol, resorcinol, pyrogallol, and phloroglucinol, which have been found to be toxic to ruminants^49^. To draw conclusive inferences and provide reliable recommendations to the animal feed industry, further investigation into mechanism in which tannin chemical composition affects rumen microbial community and its effect on ruminal fermentation is necessary.

### No significant effect of rutin addition on methane and gas production

Quercetin is one of the most investigated polyphenols from plant origin due to its antioxidative and anti-inflammatory properties. Compared to other quercetin glycosides, rutin (quercetin-3-*O*-rutinoside) is one of the most commonly found quercetin glycosides, and also the most readily available source of quercetin in the purified form^54^. Rutin is the flavanol glycoside between quercetin and the disaccharide rutinose (*α*-l-rhamnopyranosyl-(1 → 6))-*β*-d-glucopyranose)^55^. A study by Seradj et al.^56^ using a commercial flavonoid blend (Bioflavex R comprising neoeriocitrine, naringine, isonaringine, hesperidine, neohesperidine and poncirine) found that it reduced CH_4_ production in vitro by negatively affecting the hydrogenotrophic methanogenic archaeal population. Flavonoids or their degradation products could depress the microbial population by inhibiting their cytoplasmic membrane function or cell wall and nucleic acid synthesis^56^. Contrary to our initial assumption, rutin addition to the grass substrate did not significantly affect both both GP and CH_4_ production in this study. This is in concurrence with the studies by Oskoueian et al.^57^ and Nørskov et al.^58^, where rutin was found to have no effect on CH_4_ reduction compared to other tested flavonoids such as quercetin and naringin. The lower bioactivity of rutin compared to quercetin could be attributed to the presence of two sugar moieties in rutin which could reduce its bioactivity^58^.

Studies have shown that flavonoids such as rutin and naringin are readily degraded (100%) in the rumen environment, and their degradation products could act as alternative carbon source for rumen microbial population^28,57,58^. However, the increase in GP with rutin addition was not significant compared to control (only grass substrate) in the present study. Additionally, the effect of rutin addition in the presence of tannin extracts on CH_4_ production from the substrate was also analysed, as both of these polyphenolic compounds have been proposed to exert antimethanogenic activity in ruminants^59^. These compounds typically co-exist in the plant matrix and limited information is available on their associative effects. However, as seen for sole rutin addition, the addition of rutin to tannin extracts did not have a significant effect on the measured fermentation parameters. There was a trend of increase in both GP and CH_4_ production when rutin was added with big trefoil and sulla extracts (10 + 10 g/kg DM) compared to sole tannin extracts at 20 g/kg DM. However, further research is needed to understand their effect on rumen microbial populations and how these dynamics may differ in the long-term.

## Conclusion

Tannin extracts from different plant species analyzed in this study were able to reduce the CH_4_ production in vitro compared to the grass control. However, it was generally difficult to achieve significant reductions in CH_4_ emissions without simultaneous reductions in digestibility, especially from those with PD-rich PAs. Salad burnet, which is rich in HTs, and birdsfoot trefoil (PC-rich PAs) were better suited to reduce in vitro CH_4_ production without compromising forage digestion. This suggests that, apart from tannin concentration, the effectiveness of these extracts depends on their compositional characteristics, which rely highly on the species the extracts are derived from. The effect of inclusion of rutin both alone or in combination with tannins generally had a low effect, indicating that matrix effects may be of less relevance compared to the tannin concentration and composition.

Simultaneously, the efficacy of tannins to reduce CH_4_ from the substrate could also be influenced by the composition and digestibility of the diet. The absence of studies on tannin supplementation with wide range of basal diets can further impede their utilisation in livestock production systems. Therefore a systematic assessment of these extracts, including their effects on the rumen microbiome, coupled with in vivo studies, is necessary to establish the ideal supplementation that can effectively reduce CH_4_ production while maintaining optimal rumen fermentation.

## Methods

### Plant samples for tannin extracts

Four forage species were selected with diverse range of tannin composition and structural characteristics based on the previous analysis conducted by Verma et al.^33^. The selected species were birdsfoot trefoil (*Lotus corniculatus cv.* Rocco), big trefoil (*Lotus pedunculatus* cv. Lot 29), salad burnet (*Sanguisorba minor* cv. PI 308861), and sulla (*Hedysarum coronarium* cv. Sudda). The plant seeds were aquired from IPK Leibniz Plant Genetics and Crop Plant Research, Gatersleben. The plants were grown under controlled greenhouse conditions as described previously in Verma et al.^34^. Briefly, the plant samples were harvested three times and separated into different plant organs. The samples were freeze dried and milled using a ZM 200 centrifugal mill with a sieve size of 0.25 mm (Retsch, Haan, Germany), and were stored at − 80° C until further analysis. The experimental research on plants adheres to applicable institutional, national, and international guidelines and regulations.

### Tannin extraction and purification

Tannin extracts were prepared from the pooled leaf samples as described in Verma et al.^33^. The samples were extracted by weighing 0.5 g of leaf material in a 50 mL tube and adding 45 mL of 80% acetone/water (80:20, v/v) solution. The samples were vortexed and then extracted overnight on a planary shaker at 4 °C. The mixture was then centrifuged at 13,500×*g* for 15 min to obtain the extract. The extraction step was repeated for two additional times for a period of three hours using the identical setup to maximise the recovery. The extracts obtained after the extractions were concentrated to water-phase at 30 °C with the rotary evaporator and aqueous extracts were lyophilized.

Approximately 3–4 g of freeze-dried extracts were dissolved in 15 mL of water. The extracts were filtered with 0.45 μm polytetrafluoroethylene (PTFE) filters and were then loaded on a Sephadex LH-20 column (40 × 4.8 cm, Cole-Parmer, Vernon Hills, IL, USA) with a solvent gradient as described by Salminen and Karonen^26^. The samples were first eluted with water (500 mL) followed by methanol/ water mixture (1:1, v/v) solution, and then with the increasing concentrations of acetone/water (3:7, 1:1, and 4:1, v/v). The organic solvents from each fraction were simultaneously evaporated with the rotary evaporator and the remaining aqueous extracts were freeze-dried. The acetone fractions were subsequently analysed with UPLC–MS/MS to determine the purity and tannin composition of the extracts.

## Analysis of tannin extracts

Purified tannin extracts obtained after Sephadex purification were analysed for their polyphenolic composition and PA structural features according to Engström et al.^60^. 10 mg of freeze-dried extracts were dissolved in 1 mL of ultrapure water and vortexed for 5 min. The samples were filtered with a 0.20 μm PTFE filters, and 50 μL of it was transferred into the UPLC vial. Acquity UPLC system (Waters Corp., Milford, MA, USA) interfaced to a Xevo triple-quadrupole tandem mass spectrometer (Waters Corp., Milford, MA, USA) was used for this analysis with the group-specific 2D fingerprinting analyses of different tannin sub-groups as described in Engström et al.^60^, Engström et al.^61^, and Salminen^62^. The system was equipped with autosampler, Acquity UPLC BEH Phenyl column (1.7 μm, 2.1 mm × 100 mm, Waters Corp., Wexford, Ireland), and diode array detector. The flow rate of the eluent was set to 0.5 mL/min and it comprised two solvents, acetonitrile (A) and 0.1% aqueous formic acid (B). The following gradient profile was applied for the two solvents: 0–0.5 min, 0.1% A (isocratic); 0.5–5.0 min, 0.1–30% A (linear gradient); 5.0–8.0 min, and 30–45% A (linear gradient); 8.0–11.5 min, column wash, and stabilization. The data was recorded from 0 to 6 min for UV–Vis (190–500 nm) and MS (m/z 100–2000). Negative electrospray ionization was used with the following specifications; capillary voltage: 2.4 kV, desolvation temperature: 650 °C, source temperature: 150 °C, flow rate of desolvation and cone gas (N2): 1000 and 100 L/h, respectively, and collision gas: argon (0.15 mL/min).

### Substrate preparation and Hohenheim gas test

Purified tannin extracts along with rutin were tested for their antimethanogenic potential at varying concentrations (0 (Control), 10, 20 and 30 g/kg DM) using tannin-free perennial ryegrass as a basal substrate. Perennial ryegrass was harvested at vegetative stage, freeze-dried and ground to pass a 1-mm sieve (Retsch GmbH, ZM 100). The substrate was analysed according to standard protocols of Association of German Agricultural Analytic and Research Institutes (VDLUFA). The digestibility (method 6.6.1) of the substrate as well as the concentrations of CP (method 4.1.1), NDF (method 6.1.1), fat (method 5.1.1), ADF org (method 6.5.2) and crude ash (method 8.1) in the substrate were determined^63^. Metabolisable energy and NEL were estimated according to GfE^64^.

In vitro CH_4_ and GP was measured using Hohenheim gas test as described in Menke and Steingass^32^ and Verma et al.^34^. Grass samples with different extracts (200 ± 1 mg) in different concentrations, were added to 100 mL calibrated glass syringes (Haeberle Labortechnik, Lonsee-Ettlenschieß, Germany). Each sample was run in triplicates on two different days amounting to 6 replicates per sample. In order to standardize each run, four blanks (syringes without plant material), hay, and concentrate standards (3 replicates) (with known GP) from the Institute of Animal Science, University of Hohenheim, Germany were used as a reference. The ruminal fluid for the analysis was collected from two ruminally-cannulated, non-lactating crossbred heifers (Jersey × German Black Pied) with an average body weight of 565 ± 29 kg, prior to morning feeding. The animals were fed a ration comprising grass hay (3 kg) and concentrate (3 kg), divided into 2 meals (7 a.m. and 4 p.m.). After collection, ruminal fluid was filtered using cheese cloth before transferring into prewarmed insulated flask, and was immediately transported to the lab. Subsequently, it was mixed with freshly prepared buffer solution in 1:2 ratio (v/v). The mixture was continuously stirred and flushed with CO_2_ while maintained at 39 °C in a water bath. The buffered ruminal fluid (30 ml) was added to the syringes containing the samples, and syringes were placed in the incubator (39 °C) with the rotor set for a period of 24 h. Gas and CH_4_ production were measured at time intervals of 8 and 24 h. The CH_4_ concentration in the gas produced (MP) was measured with infrared spectrometer (Methan AGM 10, Firma Sensors Europe, Ratingen, Germany). Before taking the measurements, the spectrometer was calibrated with nitrogen gas (zero point) and a gas mixture of CH_4_ and CO_2_ (60:40) which were used as standards. Measured GP volume from the samples after 24 h was corrected with an average of the factors obtained from hay and concentrate standards and for GP coming solely from rumen inocula (blanks). The factors were calculated based on the measured and the targeted GP values of 45.9 mL/200 mg DM and 65.1 mL/200 mg DM after 24 h incubation of hay and concentrate standards, respectively. The parameters, OM digestibility, ME and NEL, for the samples were calculated using the equations mentioned in Menke and Steingass^32^ as follows:

$$\text{OMD }\left(\text{\%}\right)=14.88 + 0.889\text{ G}24 + 0.45\text{ CP }+ 0.0651\text{ CA}$$

$${\text{ME }}\left( {{\text{MJ}}/{\text{kg DM}}} \right)\, = \,{1}.{24}\, + \,0.{\text{1457 G24}}\, + \,0.00{7}0{\text{ CP}}\, + \,0.0{\text{224 EE}}.$$

$${\text{NEL }}\left( {{\text{MJ}}/{\text{kg DM}}} \right)\, = \, - 0.{22}\, + \,0.{1}0{\text{62 G24}}\, + \,0.00{\text{48 CP}}\, + \,0.0{\text{132 EE}}.$$

where G24 is the total gas production in 24 h (ml/200 mg DM); CP (% DM) is crude protein; CA (% DM) is crude ash and EE (% DM) is ether extract.

### Statistical analysis

The statistical analyses and graphical visualizations were done using R programming software^65^. The effect of different polyphenol extracts (tannin extract from different species and rutin) and their inclusion rate in the control was analyzed using linear mixed model using the “nlme” package in R. The statistical model included species of extract, fraction, concentration, and their interaction effect as fixed factors, and replicates nested in the date of experiment as random factor. Graphical residual analysis was conducted, based on that data was assumed to follow normal distribution and exhibited heteroscedasticity. Subsequently, ANOVA and multiple contrast tests were performed to evaluate the significance of the influence factors at different levels, and Dunnett’s test was used to assess the significant differences between the control and the tested extracts. Statistical significance of the tested treatments was established at a *p* value of less than 0.05.

In order to establish a dose–response relationship between the inclusion rate of the extracts and the tested fermentation parameters, GP, CH_4_, and MP, the initial model was modified where concentration was included as a quantitative factor. Source of extract and interaction between source and concentration was used as a fixed factor with replicates nested in the date of experiment as random factor in the new model. Based on this model, promising extract sources were identified by assessing the significant differences across slopes of these sources. Model efficiency was evaluated based on adjusted R^2^ (coefficient of determination), Nash–Sutcliffe model efficiency coefficient test, and the root mean square error.

## Data Availability

All data analysed during this study are included in this published article.
